# An octopamine-glutamate cotransmitting neuron gates aggressive escalation through layered inhibition in *Drosophila*

**DOI:** 10.1126/sciadv.aec4153

**Published:** 2026-08-14

**Authors:** Antoine Prunier, Lewis M. Sherer, Mariana Da Silva, Samantha Chong, Léa Bihl, R. Steven Stowers, Sarah J. Certel, Séverine Trannoy

**Affiliations:** ^1^ Research Center for Animal Cognition, Integrative Biology Center, Toulouse University, Toulouse, France.; ^2^ Department of Neuroscience, Brown University, Providence, RI, USA.; ^3^ Division of Biological Science, Neuroscience Program, University of Montana, Missoula, MT, USA.; ^4^ Barnard College, New York City, NY, USA.; ^5^ Department of Microbiology & Immunology, Montana State University, Bozeman, MT, USA.

## Abstract

Aggression is an evolutionarily conserved behavior essential for survival and reproduction, yet escalation to high-intensity forms entails substantial metabolic costs and injury risks, necessitating precise neural control. Using *Drosophila melanogaster*, we uncovered a multilayered inhibitory circuit that constrains aggressive escalation. This circuit involves a cotransmitting octopamine-glutamate ventral paired medial 4 (VPM4) neuron and its downstream GABAergic target, MBON-11. Neurotransmitter-specific manipulations reveal that octopamine and glutamate release from VPM4 is independently regulated by presynaptic OAα2R and mGluR receptors, providing transmitter-specific feedback. Postsynaptically, glutamate inhibits the approach-promoting MBON-11 neuron via GluClα receptors, restraining transitions to high-intensity aggression and supporting a role in approach/avoidance behaviors. Furthermore, the Rdl GABAergic receptor within MBON-11 neurons provides rapid inhibitory feedback, creating an additional layer of regulation. Together, these findings reveal a circuit architecture in which cotransmission and inhibitory feedback loops form a layered inhibition mechanism that continuously constrains escalation, indicating that aggression intensity is actively regulated to align behavior with context and cost-benefit trade-offs.

## INTRODUCTION

Aggression represents a high-stakes social behavior observed across species, essential for securing vital resources such as territory, food, and mates, as well as for self-defense (*1*). Although successful fights often confer a substantial fitness advantage, advancing from ritualized displays to direct physical engagement carries risks of injury and elevated metabolic demand even for a winner (*2*, *3*). As such, the initial reliance on threat displays before physical escalation represents an adaptive strategy to minimize such risks. Although decades of research in numerous systems have identified key neurotransmitters, neuromodulators, and neuropeptides that promote aggression, the molecular mechanisms controlling escalation and inhibition of aggression remain poorly understood (*4*). Using *Drosophila melanogaster*, we uncover a sophisticated inhibitory mechanism for controlling high-intensity aggression, comprising an octopamine (OA)-glutamate cotransmitting neuron and GABAergic interneuron, that operates through three distinct mechanisms: (i) transmitter-specific presynaptic feedback regulating OA and glutamate signaling, (ii) GluClα-mediated inhibition of the GABAergic MBON-11 interneuron, and (iii) γ-aminobutyric acid type A (GABA_A_) feedback loops controlling MBON-11 output.

Understanding the neural control of aggression requires examining how different signaling molecules work together to regulate behavior. While neuromodulatory signaling by neuropeptides and biogenic amines has long been recognized as crucial for behavior regulation (*5*–*7*), cotransmission of fast-acting transmitters with slower-acting neuromodulators is also emerging as a fundamental mechanism of neuronal communication (*8*, *9*). Our previous work revealed that most OA neurons, the invertebrate analog of norepinephrine (NE), also express the vesicular glutamate transporter (VGLUT) (*10*), which is required for transporting glutamate into synaptic vesicles, and that within this OA-glutamate cotransmitting subpopulation, OA and glutamate signaling is required to support aggressive behavior (*10*). However, dual transmission is likely not a uniform property: The same two transmitters may be used in distinct ways across neurons and synapses, so linking cotransmission to the circuit function requires moving beyond a subpopulation to determining how individual cotransmitting neurons engage their downstream partners. For example, the dual-transmitter capacity can shape behavioral circuits through temporal control of postsynaptic responses, selective targeting of receptor populations, and rapid feedback via autoreceptors (*8*, *11*). Understanding neuron-specific cotransmission properties is therefore essential for determining how aggression circuits flexibly tune behavioral intensity depending on internal and external states.

The fruit fly *D. melanogaster* provides an ideal model system for investigating mechanisms that control escalated aggression (*6*, *12*). While both *Drosophila* males and females demonstrate aggressive behaviors (*13*), male fights, in particular, progress through stereotypical stages (*12*), ranging from low-intensity encounters to mid-intensity lunges and, lastly, to high-intensity boxing. The latter plays a crucial role in forming dominance relationships that enhance access to resources and gain fitness advantages (*14*, *15*). The rare occurrence of high-intensity boxing bouts (*12*) suggests that escalation depends on a number of variables including resource value, internal state, or past experience that justifies the added metabolic expense. Among the OA-glutamate cotransmitting neuron subpopulation that might couple resource value to behavior are the ventral paired medial 4 (VPM4) neurons (*16*), previously shown to integrate internal state and gustatory cues to control feeding behavior (*17*, *18*) and thus may couple resource value to behavior. Likewise in mice, a population of locus coeruleus neurons co-releases NE and glutamate, and this cotransmitter signaling is contributing to fear-induced feeding suppression (*19*). These findings led us to hypothesize that VPM4 neurons, using OA-glutamate cotransmission analogous to NE-glutamate signaling in locus coeruleus neurons, function to integrate crucial factors, such as resource value and internal state, to control the escalation of aggression.

Through systematic manipulation of VPM4’s cotransmitter capabilities, we uncovered an elegant inhibitory system controlling aggression escalation. Our findings demonstrate that VPM4 neurons regulate high-intensity boxing through both neurotransmitters: OA suppresses male-male courtship and contributes to the modulation of aggression, while glutamate release inhibits the GABAergic approach–promoting interneuron mushroom body output neuron-11 (MBON-11) (*16*) [synonyms include MVP2 (*20*) and MBON-γ1pedc>α/β (*16*)] via the inhibitory ligand-gated chloride channel GluClα to gate or restrain high-intensity boxing. This regulation involves multiple feedback layers: Presynaptic metabotropic glutamate receptor (mGluR) and OAα2R receptors provide transmitter-specific feedback for VPM4, while GABA_A_ receptors on MBON-11 create both rapid and sustained negative feedback control. Unexpectedly, we found that increased MBON-11 GABAergic signaling escalates aggression. As previous studies demonstrated that MBON-11 promotes approach behavior by inhibiting avoidance circuits (*20*–*22*), we hypothesize that MBON-11 activity may similarly inhibit avoidance and, thus, retreats within the fight context, leading to increased high-intensity behaviors. An inhibition of retreat, a survival strategy that normally limits escalation, could explain the high-intensity aggressive encounters we observed. Collectively, our results demonstrate how cotransmission and layered inhibition create a dynamic control system for behavioral escalation, offering new insights into how neural circuits regulate adaptive and pathological aggression across species.

## RESULTS

### Cotransmitting VPM4 neurons are required for high-intensity aggression

**Fig. 1.**
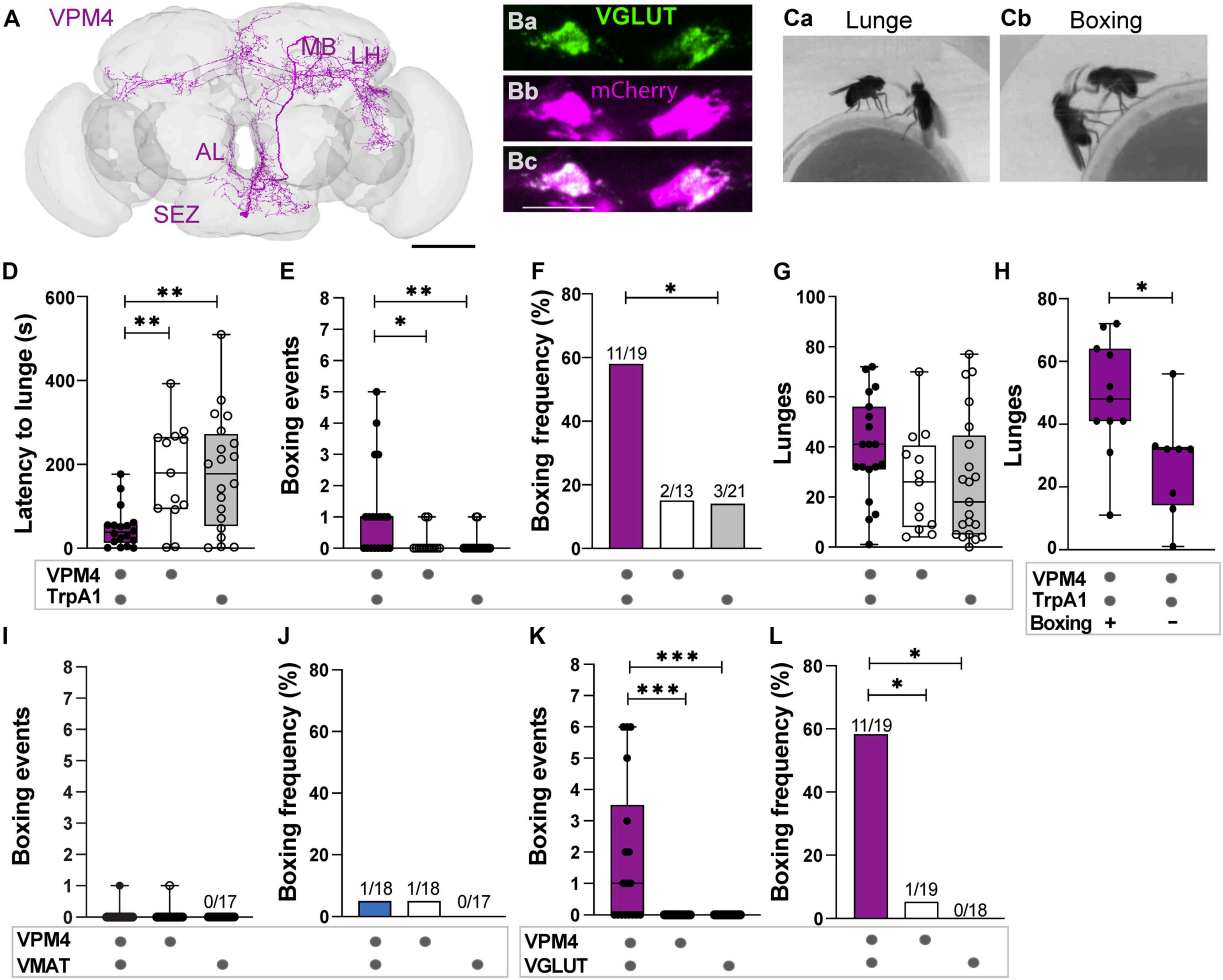
Glutamatergic signaling from VPM4 promotes escalation of male aggression. (**A**) Connectome-based representation of the VPM4 neuron. The cell bodies are located in the SEZ with axon projections to the Antennal Lobes (ALs), the Lateral Horn (LH), and the Mushroom Body (MBs). The scale bar represents 50 um. (**B**) VGLUT expression (anti-VGLUT, green) in VPM4 neurons (MB113C-gal4; UAS-mCherry). (**C**) Aggressive lunge behavior by the male on the right (Ca) and two males boxing (Cb). (**D**) Latency to lunge decreased upon thermal activation of VPM4 neurons (*VPM4>dTrpA1*) [Kruskal-Wallis, *H* = 14.01, *P* = 0.0009; Dunn’s test with *VPM4/+* (*P* = 0.003) and *+/dTrpA1* (*P* = 0.005)]. (**E**) The number of boxing events increased upon VPM4 activation (*VPM4>dTrpA1*) [Kruskal-Wallis, *H* = 14.13, *P* = 0.0009; Dunn’s test with *VPM4/+* (*P* = 0.008) and *+/dTrpA1* (*P* = 0.002)], as well as (**F**) boxing frequency [generalized linear model with a binomial distribution = 11.457, df = 2, *P*= 0.003; Tukey post test with *VPM4/+* (*P* = 0.037) and with *+/dTrpA1* (*P* = 0.018)]. (**G**) Across all VPM4-activated male pairs, the number of lunges did not differ from controls (Kruskal-Wallis, *H* = 5.711, *P* = 0.0575). (**H**) The lunge number was higher in *VPM4>dTrpA1* male pairs that exhibited boxing compared to those that did not box (Mann-Whitney *U* = 17, *P* = 0.0253). (**I**) Elevated levels of VMAT in VPM4 neurons did not affect the number of boxing events (Kruskal-Wallis, *H* = 0.963, *P* = 0.618) or (**J**) the boxing frequency (generalized linear model with a binomial distribution = 0.0023, df = 2, *P* = 0.999). (**K**) Elevated vGLUT expression in VPM4 neurons resulted in a significant increase in both the number of boxing events [Kruskal-Wallis, *H* = 23.78, *P* < 0.0001; Dunn’s tests with *VPM4/+* and *+/VGLUT* (*P* < 0.0001)] and (**L**) boxing frequency [generalized linear model with a binomial distribution = 19.264, df = 2, *P* = 6.559 × 10^−05^; Tukey post test with *VPM4/+* (*P* = 0.012) and with *+/dTrpA1* (*P* = 0.015)]. ns, *P* > 0.05; *P ≤ 0.05; **P ≤ 0.01; ***P ≤ 0.001.

Located within the subesophageal zone (SEZ) are the OA-glutamate VPM4 neurons that synapse onto sugar-sensitive gustatory receptor neuron terminals, amplifying their sucrose responses (*17*). This amplification not only stimulates feeding but may also elevate resource stakes. The presence of a sucrose patch in the fighting arena significantly increases male-male aggression, an effect abolished when specific gustatory receptor neurons are silenced (*23*). VPM4 neurons relay this sensory information to higher brain centers via projections to the antennal lobe (AL), lateral horn (LH), and γ-lobe of the mushroom body (MB) [Fig. 1A; FlyWire (*24*)] (*16*). Thus, their anatomical and functional connectivity, combined with their coexpression of OA and glutamate transporters (Fig. 1B) (*10*), positions VPM4 neurons to integrate resource value with aggressive drive, potentially enabling rapid, context-dependent behavioral flexibility.

To initially test this hypothesis, we expressed the thermosensitive cation channel TrpA1 (*25*) under the control of the VPM4-specific MB113C-split-Gal4 driver (*VPM4>dTrpA1*) (*16*) and transiently activated these neurons via temperature shift. Each fighting chamber contained small cups of standard fly food (cornmeal, agar, sucrose, and yeast), creating a tangible territory and ensuring sucrose-driven activation of VPM4. Aggression was quantified by scoring the latency to the first lunge, the number of mid-intensity lunges (Fig. 1Ca), and the number of high-intensity boxing (when males rear up and strike with their forelegs) (Fig. 1Cb) (*12*, *26*). We focused on males, as they display high-intensity boxing, an escalation pattern that often emerges when neither opponent retreats during lower-intensity behaviors. Females do exhibit aggression, but their fights have not been reported to escalate (*13*).

Acute VPM4 activation significantly elevated male aggression, demonstrated by a rapid onset of aggressive behavior (reduced latency to lunge; Fig. 1D) and an increase in both the number and frequency of high-intensity boxing events (Fig. 1, E and F). A total of 58% (11 of 19) of pairs exhibited at least one boxing bout (Fig. 1F), although the total number of lunges did not differ between experimental and control groups (Fig. 1G). However, within the *VPM4>dTrpA1* experimental group, males in fights that included at least one boxing event displayed significantly more lunges than those in fights without boxing (Fig. 1H). At permissive temperature, aggression levels remained unchanged (fig. S1). Thus, VPM4 neuron activity is sufficient for males to engage in high-intensity aggressive behaviors.

To dissect the contributions of OA versus glutamate in aggression escalation, we selectively increased the expression of the vesicular monoamine transporter (VMAT) or VGLUT to increase neurotransmitter release. VMAT overexpression (*VPM4>VMAT*) had no effect on the number or frequency of boxing events (Fig. 1, I and J). Vesicular transporter overexpression can increase monoamine packing capacity, yet behavioral output may remain unchanged if vesicle release probability rather than vesicular filling is the limiting step for transmitter output in this circuit (*27*). In contrast, VGLUT overexpression (*VPM4>VGLUT*) significantly increased both the number and frequency of boxing events compared to controls (Fig. 1, K and L). Specifically, 58% of *VPM4>VGLUT* male pairs exhibited boxing, whereas this behavior was rare in control groups (5 and 0%, respectively). These results indicate that glutamate release from VPM4 neurons is key to promoting escalation of male aggression, possibly by relaying gustatory cues or food-related value to downstream targets, where the excitatory or inhibitory outcome would depend on postsynaptic receptor composition.

### Presynaptic OA receptors provide inhibitory feedback to constrain aggression

**Fig. 2.**
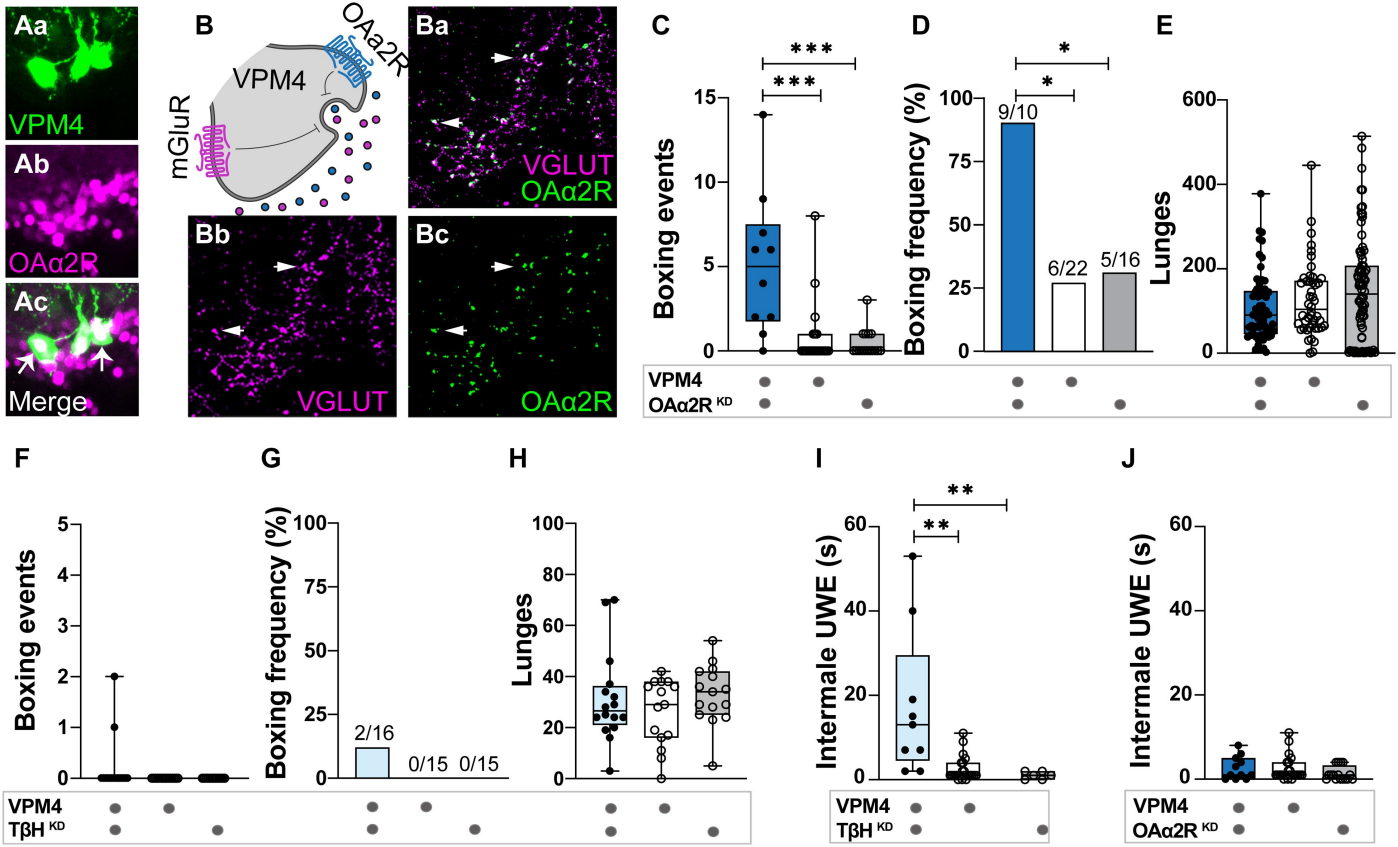
Aggression is constrained by presynaptic OA receptors. (**A**) *MB113C-split-Gal4* and *OAa2R-LexA* together with UAS-6XGFP and LexAop-6XmCherry reporters demonstrated that OAα2R (magenta) is expressed in VPM4 neurons (green). (**B**) Using the *MB113c-split-Gal4* driver to restrict conditional expression to VPM4 neurons, OAα2R-40xFLAG (green) localized to VPM4 presynaptic terminals marked by VGLUT-6XV5 (magenta). (**C**) The number of boxing events was significantly higher in *VPM4>OA*α*2R^KD^* males compared to controls [Kruskal-Wallis, *H* = 17.36, *P* = 0.0002; Dunn’s test with *VPM4/+* (*P* = 0.0003) and with +/*OA*α*2R*^KD^ (*P* = 0.0007)]. (**D**) *VPM4>OA*α*2R^KD^* males displayed higher boxing frequency than genetic controls [generalized linear model with a binomial distribution = 13.044, df = 2, *P* = 0.0015; Tukey post test with *VPM4/+* (*P* = 0.016) and with *+/OA*α*2R^KD^* (*P* = 0.031)]. (**E**) The number of lunges was not significantly increased in *VPM4>OA*α*2R^KD^* males compared to genetic controls (Kruskal-Wallis, *H* = 3.664, *P* = 0.16). (**F**) Statistical differences between controls and *VPM4>T*β*H^KD^* males did not occur in boxing events [Kruskal-Wallis, *H* = 1.805, *P* = 0.406; Dunn’s test with VPM4/+ (*P* = 0.875) and with *+/T*β*H^KD^* (*P* = 0.999)], (**G**) boxing frequency (Fisher’s exact test for count data, *P* = 0.318), and (**H**) lunge number [Kruskal-Wallis, *H* = 3.833, *P* = 0.148; Dunn’s test with *VPM4/+* (*P* = 0.999) and with *+/T*β*H^KD^* (*P* = 0.275)]. (**I**) *VPM4>T*β*H^KD^* showed increased intermale courtship compared to controls [Kruskal-Wallis, *H* = 15.46, *P* = 0.0004; Dunn’s test with *VPM4/+* (*P* = 0.00013) and with *+/T*β*H^KD^* (*P* = 0.0025)]. (**J**) On the contrary, *VPM4>OA*α*2R^KD^* males did not show elevated intermale courtship (Kruskal-Wallis, *H* = 3.163, *P* = 0.2056). ns, *P* > 0.05; *P ≤ 0.05; **P ≤ 0.01; ***P ≤ 0.001.

Given the energetic cost of high-intensity aggression, the nervous system must implement mechanisms that prevent excessive neuromodulatory output. One such mechanism involves presynaptic autoreceptors, which detect a neuron’s own transmitter release and inhibit further output, thereby stabilizing neural activity (*28*, *29*), consistent with presynaptic feedback limiting release probability in this circuit. In *Drosophila*, OAα2R, an ortholog of vertebrate α2-adrenergic receptors, reduces OA release through inhibition of adenosine 3′,5′-monophosphate synthesis (*30*, *31*), suggesting that it could function as a presynaptic inhibitory autoreceptor. We first confirmed that OAα2R is expressed in VPM4 neurons using an *OA*α*2R-lexA* driver in combination with *VPM4-Gal4* and separate fluorescence reporters (Fig. 2A). We then examined the subcellular localization of OAα2R by generating a conditional OAα2R allele containing a multimerized V5-epitope tag (*32*). When conditionally expressed in VPM4 neurons, OAα2R-V5 localized to VPM4 terminals marked with VGLUT, indicating enrichment at presynaptic sites and providing support for a potential presynaptic inhibitory autoreceptor function (Fig. 2B).

Previous manipulations that broadly increased OA availability, such as dietary OA supplementation; overexpression of *Tyramine* β*-hydroxylase* (*T*β*H*), the enzyme required for OA synthesis; or global activation of OA neurons using TrpA1, did not significantly alter aggression levels (*33*–*35*). Although a subset of OA neurons in the SEZ has been shown to modulate aggression (*36*), it remained unclear whether increased OA signaling from a single neuronal pair could influence aggression. Given its previously described inhibitory role (*30*, *31*) and its presynaptic localization (Fig. 2B), we hypothesized that reducing OAα2R expression in VPM4 would weaken OA-dependent negative feedback and therefore increase OA output when VPM4 is active, mimicking *VGLUT* overactivation effects. Consistent with this idea, knocking down OAα2R in VPM4 neurons (*VPM4>OA*α*2R^KD^*) significantly increased both the number and frequency of high-intensity boxing events (Fig. 2, C and D) without affecting lunge number (Fig. 2E), indicating a selective effect on escalation. These findings are consistent with OAα2R contributing to inhibitory feedback in VPM4 neurons that limits OA release and therefore gates aggressive escalation. However, as OA release was not directly measured, we cannot exclude additional mechanisms, including potential postsynaptic actions of OAα2R (*37*, *38*).

To directly test whether OA from VPM4 is required for aggressive behavior, we reduced OA synthesis by knocking down TβH in VPM4 neurons (*VPM4>T*β*H^KD^*). Unlike OAα2R knockdown, which is predicted to increase OA release, TβH knockdown is expected to reduce OA production, yet boxing events (Fig. 2F), boxing frequency (Fig. 2, E to G), and lunge number (Fig. 2H) were comparable to genetic controls. Unlike broad reductions in OA availability, which decrease aggression initiation (*36*), our findings suggest that synapse-specific regulation of OA output, supported by OAα2R knockdown (Fig. 2, C to E) and consistent with VMAT spatial segregation in VPM4 neurons (*10*), controls escalation rather than total OA levels.

The same two manipulations produced a notable difference in courtship behavior. OA is crucial for insect reproduction (*39*–*41*), and we have shown that OA suppresses male-male courtship during aggressive encounters (*34*, *35*, *42*). To assess the role of VPM4-derived OA in this behavioral context, we reduced OA synthesis by knocking down TβH in VPM4 neurons and quantified unilateral wing extensions, a key male courtship display. Males with reduced OA production (*VPM4>T*β*H^KD^*) exhibited significantly elevated unilateral wing extensions compared to genetic controls (Fig. 2I), indicating that VPM4-derived OA synthesis contributes to the suppression of male-male courtship. In contrast, reducing OAα2R expression in VPM4 neurons (*VPM4>OA*α*2R^KD^*) did not significantly affect male-male courtship (Fig. 2J), suggesting that modulation of OA feedback does not affect this behavior under these conditions. Together, these results are consistent with VPM4 contributing to dual behavioral control through distinct output mechanisms: Male-male courtship suppression depends on sufficient global OA output from VPM4, potentially acting broadly across courtship-related circuits, whereas escalation is regulated by OAα2R-dependent feedback that constrains VPM4 activity.

### Aggression is also constrained by mGluR-mediated regulation of glutamate release

**Fig. 3.**
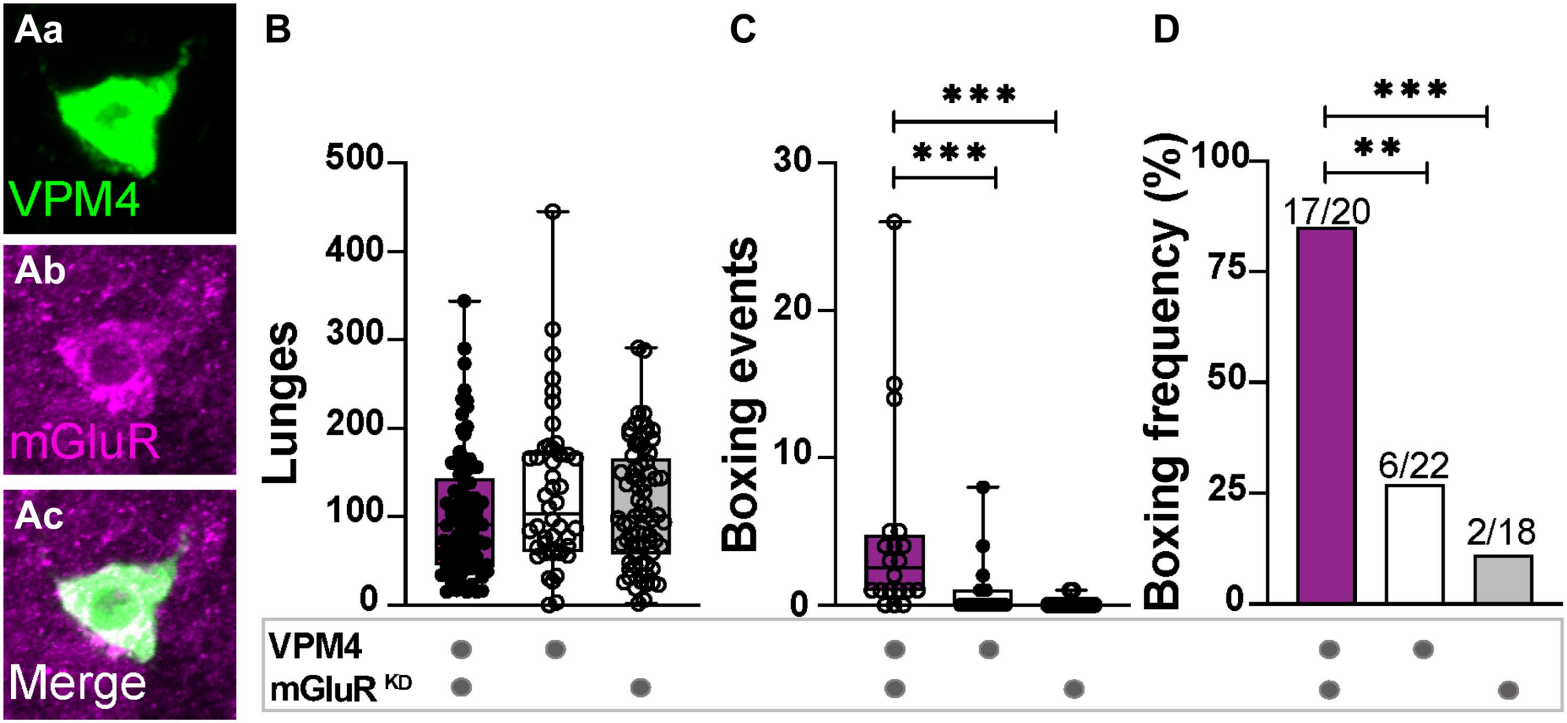
Aggression is constrained by presynaptic glutamate receptors. (**A**) mGluR expression (anti-mGluR, magenta) in VPM4 neurons (MB113C-gal4; UAS-GFP, green). (**B**) The lunge number did not increase in *VPM4>mGluR^KD^* males compared to controls (Kruskal-Wallis, *H* = 2.8, *P* = 0.2466). (**C**) Knockdown of mGluR in VPM4 increased the number of boxing events [Kruskal-Wallis, *H* = 17.20, *P* = 0.0002; Dunn’s test with *VPM4/+* (*P* = 0.0002) and with *+/mGluR^KD^* (*P* = 0.019)] and (**D**) the boxing frequency [generalized linear model with a binomial distribution = 26.255, df = 2, *P* = 0.0015; Tukey post test with *VPM4/+* (*P* = 0.0017) and with *+/mGluR^KD^* (*P* = 0.0003)]. ns, *P* > 0.05; *P ≤ 0.05; **P ≤ 0.01; ***P ≤ 0.001.

Glutamate transmission is also regulated by presynaptic autoreceptors, namely mGluRs (*43*, *44*). The single *Drosophila* mGluR is homologous to vertebrate group II mGluRs (*45*). Using a characterized monoclonal antibody (*46*), we confirmed mGluR expression in VPM4 neurons (Fig. 3A). We knocked down mGluR in VPM4 neurons (*VPM4>mGluR^KD^*) using a previously validated line (*47*) and scored aggression. This manipulation had no effect on lunge counts (Fig. 3B). However, the number of high-intensity boxing bouts significantly increased (Fig. 3B), and ∼85% of fights between *VPM4>mGluR^KD^* males involved two or more boxing bouts (Fig. 3, C and D). In rodents, administration of the selective mGluR2/3 agonist LY37926, thought to act primarily at presynaptic autoreceptors to reduce glutamate release, suppresses male aggression (*48*). These results are therefore consistent with mGluR functioning as a presynaptic autoreceptor that limits glutamate release and gates escalation, although direct measurement of glutamate release will be needed to exclude additional mechanisms. These findings support the idea that mGluR-dependent regulation of glutamate transmission may act as a brake on aggression-related circuit output, in line with established presynaptic feedback roles for group II–like mGluRs. Collectively, these experiments indicate that knocking down OAα2R and mGluR in VPM4 neurons shifts the behavioral consequences of VPM4 activity toward escalation of aggression.

### The VPM4-MBON-11 connection links sensory integration to behavioral escalation

**Fig. 4.**
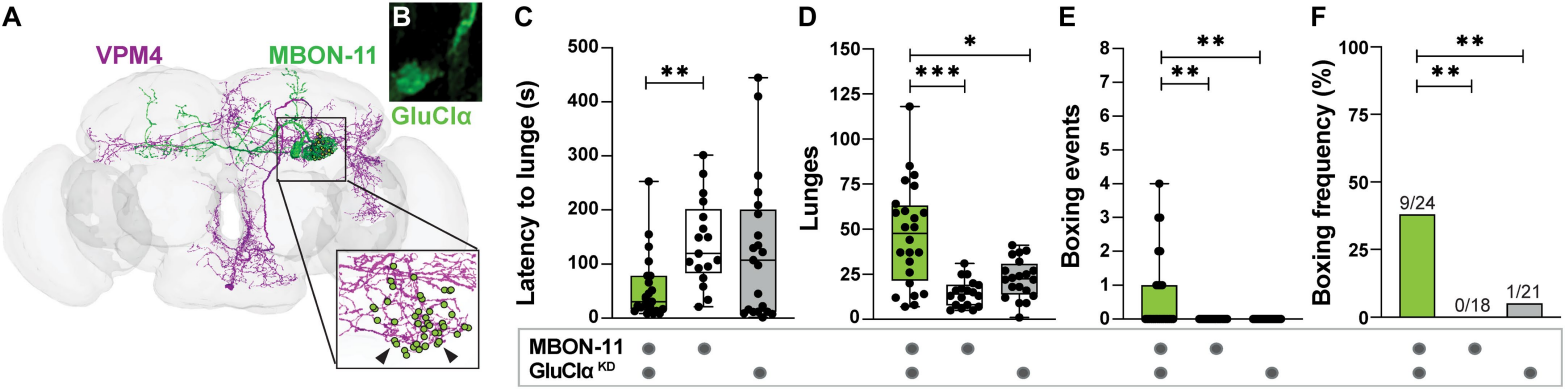
GluClα-mediated inhibition of MBON-11 limits high-intensity aggression. (**A**) Schematic representation of VPM4 and MBON-11 neurons (FlyWire). Those synaptic connections are located around the γ-lobe of the MB. (**B**) Localization of conditional GluClα-smV5 in MBON-11 (*MB112c-split-Gal4>GluCl*α*-KDRT-STOP-KDRT-smGdP-10xV5 UAS-KD*). (**C**) Knockdown of GluClα in MBON-11 (*MBON-11>GluCl*α*^KD^*) reduced latency to lunge compared to GAL4 control [Kruskal-Wallis, *H* = 9.751, *P* = 0.0076; Dunn’s test with *MBON-11/+* (*P* = 0.0057)] but not to UAS control (*P* = 0.2843). (**D**) The number of lunges is increased compared to controls [Kruskal-Wallis, *H* = 19.72, *P* < 0.0001; Dunn’s test with *MBON-11/+* (*P* < 0.0001) with *+/GluCl*α*^KD^* (*P* = 0.038)]. (**E**) The number of boxing events was higher in *MBON-11>GluCl*α*^KD^* compared to both GAL4 [Kruskal-Wallis, *H* = 16.28, *P* = 0.0003; Dunn’s test with *MBON-11/+* (*P* = 0.0022) and with +/*GluCl*α*^KD^* (*P* = 0.0015)]. (**F**) The boxing frequency is significantly higher in *MBON-11>GluCl*α*^KD^* males [Fisher’s exact test for count data, *P* = 0.0008; Holm-Bonferroni post test with *MBON-11/+* (*P* = 0.005) and with *+/GluCl*α*^KD^* (*P* = 0.011)]. ns, *P* > 0.05; *P ≤ 0.05; **P ≤ 0.01; ***P ≤ 0.001.

We next asked how VPM4 output is routed through downstream targets to implement escalation. Connectomic data from the FlyWire consortium indicate that VPM4 neurons form synaptic connections primarily with inhibitory partners [FlyWire (*23*)]. Among them, one of the top output partners is MBON-11, a GABAergic interneuron in the γ-lobe of the MB known to promote approach behavior [Fig. 4A; FlyWire (*16*, *18*, *20*, *21*, *24*)]. MBONs are functionally analogous to vertebrate basal ganglia as they process inputs from Kenyon cells in the MB, the insect’s key associative memory center (*49*). Each type of MBON is responsible for encoding the valence of a stimulus (*16*, *22*, *50*–*52*). MBON-11 specifically integrates short-term aversive memory to produce approach or avoidance behavioral outputs (*20*, *21*, *53*–*56*).

The valence of VPM4-derived glutamate signaling onto MBON-11 depends on postsynaptic receptor composition, as glutamate can mediate either excitation via *N*-methyl-d-aspartate receptors (NMDARs) or inhibition via the glutamate-gated chloride channel GluClα. Transcriptomic data indicate that both NMDARs and GluClα are expressed in MBON-11 neurons (*57*), and using an NMDAR2-Gal4 reporter line, we confirmed NMDAR2 expression in MBON-11 (fig. S2A). Using published validated RNA interference (RNAi) lines (*58*), we knocked down the expression of *NMDAR1* and *NMDAR2* in MBON-11 and did not detect an effect on aggression metrics (fig. S2), although we cannot exclude the notion that incomplete knockdown efficiency may mask a contribution of NMDAR-mediated signaling. These results raise the possibility that VPM4-derived glutamate acts through GluClα-mediated inhibition of MBON-11 to promote escalation.

A prior calcium imaging study has demonstrated that VPM4 directly inhibits MBON-11, as optogenetic activation of VPM4 neurons significantly reduced baseline MBON-11 GCaMP fluorescence (*18*). Because glutamate can mediate either excitatory or inhibitory effects depending on postsynaptic receptor composition, we next asked whether glutamate-gated inhibition contributes to this interaction. Transcriptomic data and V5-tagging allele (*59*) indicate that MBON-11 neurons express the inhibitory glutamate receptor GluClα (Fig. 4B), providing a potential mechanism for glutamatergic inhibition on MBON-11. To test this, we knocked down GluClα specifically in MBON-11 neurons using a previously characterized RNAi line (*MBON-11>GluCl*α*^KD^*) (*60*). If GluClα mediates inhibitory input from VPM4, its loss would be expected to weaken glutamate-dependent inhibition of MBON-11 and promote escalation. Consistent with this prediction, *MBON-11>GluCl*α*^KD^* males initiated fights more rapidly (Fig. 4C), exhibited increased lunge numbers (Fig. 4D), and showed a higher boxing frequency (Fig. 4, E and F). Together with prior calcium imaging data (*18*), these results are consistent with a model in which glutamatergic signaling contributes to the inhibition of MBON-11 via GluClα-dependent chloride conductance. This interpretation is independent of the VGLUT overexpression experiments, which increase the potential for glutamate release but do not define whether it is excitatory or inhibitory in this circuit context.

### GluClα-dependent effects are not recapitulated by increasing MBON-5/6 glutamatergic output

Connectome analysis indicates that MBON-11 also receives glutamatergic input from additional presynaptic partners, including MBON-5 (*24*). To test whether MBON-5–derived glutamate can modulate aggression through MBON-11, we increased the capacity for glutamatergic transmission by elevating VGLUT expression in MBON-5/6 using the MB298B-split-Gal4 line (*MBON-5/6>VGLUT*) (*16*). This manipulation did not affect fight initiation (fig. S3A), lunge number (fig. S3B), the number of boxing bouts (fig. S3C), or boxing frequency (fig. S3D). Thus, increasing glutamate release from MBON-5/6 does not phenocopy the escalation-promoting effects observed following VPM4 manipulations. One explanation is that glutamatergic inputs to MBON-11 are functionally compartmentalized: Distinct presynaptic sources may target different MBON-11 subcellular domains where inhibitory GluClα and excitatory glutamate receptors are spatially segregated, producing input-specific consequences of glutamate signaling. Consistent with this idea, our results demonstrate that VPM4 output can trigger aggression (Fig. 1F, *VPM4>TrpA1*; Fig. 1L, *VPM4>VGLUT*) but also suggest that not all glutamatergic inputs to MBON-11 produce the same behavioral outcome (fig. S3).

### Conditional activation of MBON-11 neurons triggers high-intensity aggression

**Fig. 5.**
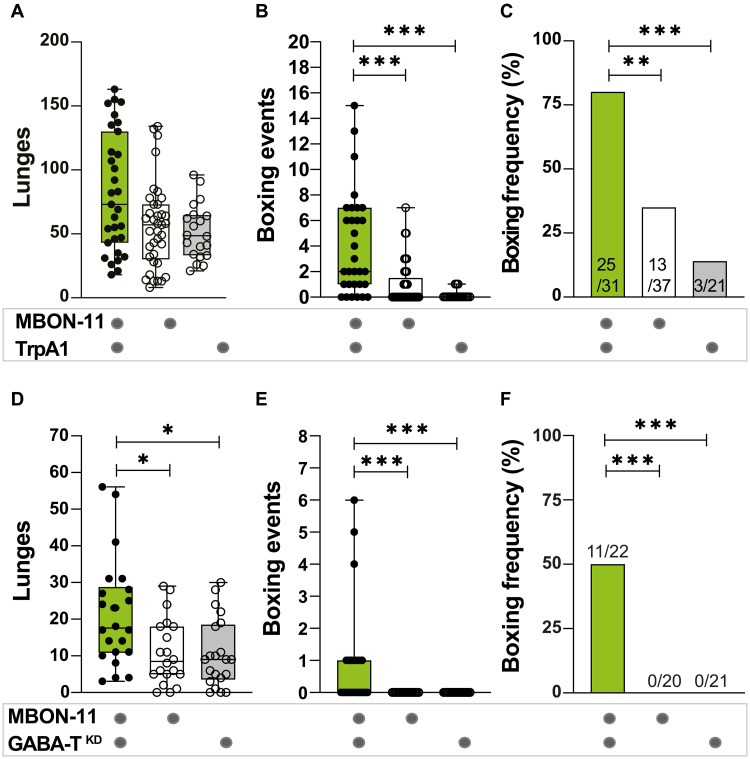
High-intensity aggression is promoted by MBON-11 activation or GABA accumulation. (**A**) Upon MBON-11 activation (*MBON-11>TrpA1*), the lunge number showed a modest genotype effect but did not differ significantly from either genetic control in post hoc comparisons [*H* = 6.604, *P* = 0.037; Dunn’s test with *MBON-11/+* (*P* = 0.076) and Dunn’s test with *+/TrpA1* (*P* = 0.093)]. (**B**) MBON-11 activation increases boxing events [Kruskal-Wallis, *H* = 30.35, *P* < 0.0001; Dunn’s test with *MBON-11/+* and with *+/TrpA1* (*P* < 0.0001)]. (**C**) The boxing frequency increased with MBON-11 activation [generalized linear model with a binomial distribution = 27.169, df = 2, *P* = 1.26 × 10^−06^; Tukey post test with *MBON-11/+* (*P* = 0.001) and with *+/TrpA1* (*P* = 0.0001)]. (**D**) The down-regulation of GABA-T within MBON-11 (*MBON-11>GABA-T^KD^*) significantly increases the number of lunges compared to controls [Kruskal-Wallis, *H* = 8.763, *P* = 0.012; Dunn’s test with *MBON-11/+* (*P* = 0.047) and with *+/GABA-T^KD^* (*P* = 0.023)]. (**E**) Number of boxing events [Kruskal-Wallis, *H* = 22.75, *P*< 0.0001; Dunn’s test with *MBON-11/+* (*P* = 0.0001); Dunn’s test with *+/GABA-T^KD^* (*P* = 0.0001)]. (**F**) The boxing frequency is also significantly higher [Fisher’s exact test for count data, *P* = 1.9991 × 10^−06^; Holm-Bonferroni post test with *MBON-11/+* (*P* = 0.0002) and with *+/GABA-T^KD^* (*P* = 0.0001)]. ns, *P* > 0.05; *P ≤ 0.05; **P ≤ 0.01; ***P ≤ 0.001.

Our findings place MBON-11 as a critical downstream node through which GluClα-mediated inhibition gates progression to aggression escalation. Given that GluClα inhibits MBON-11, a simple prediction is that activating these GABAergic neurons would inhibit their downstream neurons and reduce aggression. However, GABAergic signaling does not uniformly suppress aggression and can instead promote escalation in a circuit-dependent manner. For example, a recent study demonstrated that GABA release from mAL neurons promotes rather than suppresses aggression in *Drosophila* (*61*), while GABAergic signaling can increase aggression indirectly through modulation of serotonergic neurons in the mouse medial prefrontal cortex (*62*). In line with these findings, we found that conditional activation of the GABAergic MBON-11 (*MBON-11>TrpA1*) significantly increased high-intensity aggression, with 81% of pairs exhibiting elevated boxing behavior, while total lunges remained unchanged (Fig. 5, A to C); this effect was temperature-specific and absent under nonactivating conditions (fig. S4). Thus, MBON-11–derived GABA release is sufficient to promote escalation, consistent with effects mediated through its downstream targets. In this scenario, MBON-11 activation would drive escalation by inhibiting retreat- or avoidance-promoting targets, thereby disinhibiting aggression.

How might activation of this GABAergic neuron, typically associated with inhibition, paradoxically escalate aggression? A previous study has shown that MBON-11 regulates approach and avoidance: Its activation inhibits avoidance, biasing flies toward approach even in previously aversive contexts (*20*). In aggression, an MBON-11–driven approach bias could limit retreat and sustain a close-range interaction, promoting escalation into boxing without increasing the frequency of lunges. Consistent with this model, Kim *et al.* (*54*) found that MBON-11 is required for aversive memory formation following social defeat, and this defeat memory drives avoidance in loser males.

### GABA accumulation within MBON-11 neurons promotes aggressive escalation

To determine whether MBON-11–driven behavioral escalation is mediated specifically by its GABAergic output, we asked whether increasing GABA availability within MBON-11 could phenocopy the aggression effects of conditional MBON-11 activation. To test this, we overexpressed glutamate decarboxylase 1 (GAD1), the enzyme that converts glutamate into GABA (*MBON-11>GAD1*). Overexpression of GAD1 in MBON-11 (*MBON-11>GAD1*) did not change aggression (fig. S5). This result aligns with an earlier study showing that GAD1 is not typically rate-limiting for GABA synthesis, as its activity is constrained by posttranslational regulation and cofactor availability (*63*).

Next, we reduced GABA catabolism by knocking down GABA-T, an enzyme required for GABA degradation, within MBON-11. This led to a broad escalation of aggression: *MBON-11>GABA-T^KD^* males exhibited significantly more lunges (Fig. 5D), increased boxing events (Fig. 5E), and higher boxing frequency (Fig. 5F) than controls. In line with direct activation of MBON-11 (Fig. 5, A to C), these findings indicate that intracellular GABA accumulation within MBON-11, achieved via reduced GABA degradation, is sufficient to increase aggression.

### Blocking GABA prevents MBON-11 activation–induced high-intensity aggression

**Fig. 6.**
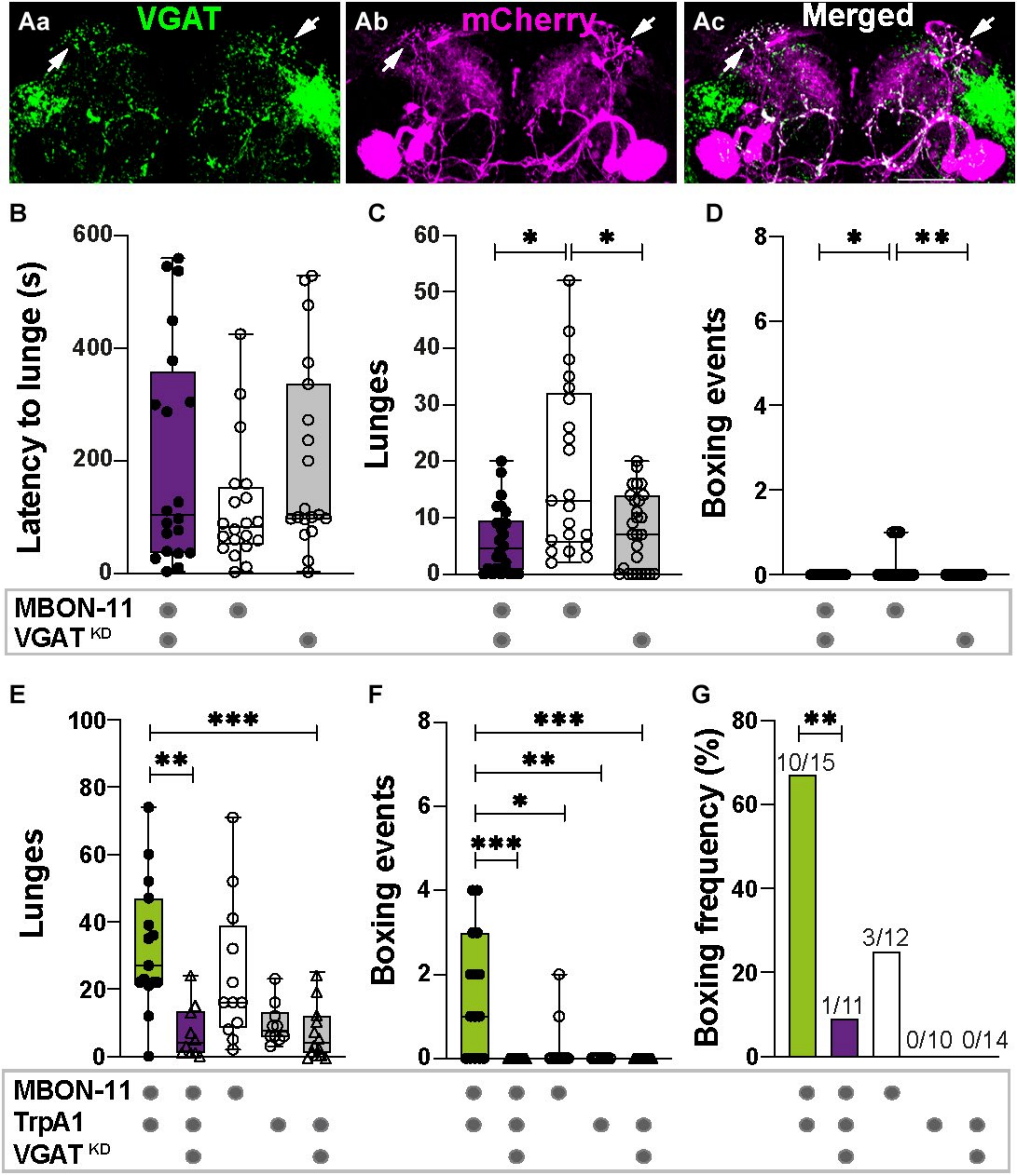
MBON-11 GABA release is recruited in a state-dependent manner during escalation. (**A**) Expression of conditional 9XV5-VGAT (green) in MBON-11 (magenta) (*MB113C-splitGal4*>*RSRT-STOP-RSRT-9XV5-vGAT*, *20XUAS-R, UAS-CD8-mCherry*). (**B**) Down-regulating the expression of VGAT in MBON-11 (*MBON-11>VGAT^KD^*) does not affect the latency to lunge (Kruskal-Wallis, *H* = 3.312, *P* = 0.191). (**C**) The number of lunges did not differ between *MBON-11>VGAT^KD^* males and controls, although the GAL4 control exhibited higher lunge counts than the other genotypes [Kruskal-Wallis, *H* = 11.25, *P* = 0.004; Dunn’s test with *+/VGAT^KD^* (*P* = 0.039); Dunn’s test with *MBON-11/VGAT^KD^* (*P* = 0.003)]. (**D**) Boxing was not observed in *MBON-11>VGAT^KD^* experimental males, and *MBON-11/+* control males exhibited more boxing events than other genotypes [Kruskal-Wallis, *H* = 11.06, *P* = 0.004; Dunn’s test with *+/VGAT^KD^* (*P* = 0.009) and with *MBON-11>VGAT^KD^* (*P* = 0.01)]. (**E**) The number of lunges decreased in *MBON-11/TrpA1; VGAT^KD^* males compared to *MBON-11/TrpA1* males (Kruskal-Wallis, *H* = 23.35, *P* = 0.0001; Dunn’s test, *P* = 0.0021). (**F**) Activating MBON-11 induced boxing events (*MBON-11>TrpA1*, green); however, when decreasing VGAT expression in parallel, boxing was abolished (*MBON-11>TrpA1; VGAT^KD^*, purple) (Kruskal-Wallis, *H* = 28.21, *P* =< 0.0001, Dunn’s test, *P* = 0.0005), as well as (**G**) the boxing frequency [Fisher’s exact test for count data, *P* = 2.585 × 10^−05^; Holm-Bonferroni post test with *MBON-11/TrpA1* (*P* = 0.005)]. ns, *P* > 0.05; *P ≤ 0.05; **P ≤ 0.01; ***P ≤ 0.001.

GABAergic transmission relies on the vesicular GABA transporter *VGAT*, which loads GABA into synaptic vesicles for Ca^2+^-dependent exocytosis. We confirmed *VGAT* expression in MBON-11 using a conditionally expressible V5-tagged variant of VGAT (Fig. 6A) (*64*). If GABA signaling increases aggression, reducing GABA release from MBON-11 via targeted knockdown of *VGAT* should reduce aggression. However, knocking down *VGAT* expression with a previously published line (*65*) did not significantly alter aggression-related behaviors. Neither the latency to lunge (Fig. 6B), the number of lunges (Fig. 6C), nor the number of boxing bouts (Fig. 6D) differed from controls, suggesting that basal MBON-11 GABA release is not required for aggression escalation under these conditions. We next considered the notion that GABAergic output from MBON-11 might only be behaviorally relevant when these neurons are actively engaged. To test this, we conditionally activated MBON-11 while simultaneously knocking down *VGAT* (*MBON-11>VGAT^KD^; TrpA1*). As expected, *MBON-11>TrpA1* males exhibited elevated levels of high-intensity aggression (Fig. 6, E to G, green). However, simultaneous disruption of GABA release in these neurons abolished this effect, as *MBON-11>VGAT^KD^; TrpA1* males no longer displayed increased boxing behavior (Fig. 6, E to G, purple versus green). These findings indicate that GABA release is recruited upon MBON-11 activation to escalate aggression.

### Rdl-dependent GABAergic signaling constrains MBON-11–mediated aggression

**Fig. 7.**
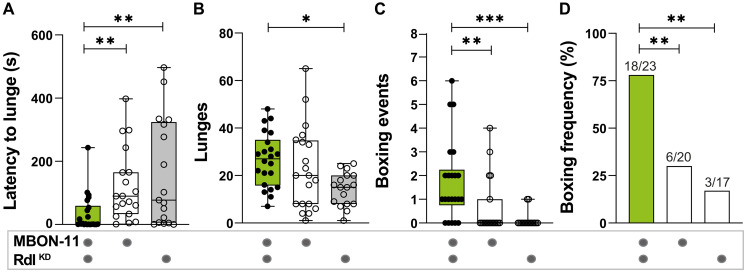
Rdl-mediated feedback limits escalation of aggression. (**A**) Knockdown of Rdl in MBON-11 neurons (*MBON-11>Rdl^KD^*) significantly shortened lunge latency as compared to controls [Kruskal-Wallis, *H* = 13.96, *P* = 0.0009; Dunn’s test with MBON-11/+ (*P* = 0.003) and with *+/Rdl^KD^* (*P* = 0.007)]. (**B**) The lunge number increased relative to the UAS control but did not differ from the GAL4 control [Kruskal-Wallis, *H* = 9.120, *P* = 0.010; Dunn’s test with *+/Rdl^KD^* (*P* = 0.008)] but not to *MBON-11/+* (Dunn’s test, *P* = 0.507). (**C**) Boxing events significantly increased [Kruskal-Wallis, *H* = 17.95, *P* = 0.001; Dunn’s test with *MBON-11/+* (*P* = 0.007) and with *+/Rdl^KD^* (*P* = 0.0002)], as well as boxing frequency (**D**) [generalized linear model with a binomial distribution = 18.213, df = 2, *P* = 0.0001; Tukey post test with *MBON-11/+* (*P* = 0.007) and with *+/Rdl^KD^* (*P* = 0.001)]. ns, *P* > 0.05; *P ≤ 0.05; **P ≤ 0.01; ***P ≤ 0.001.

To investigate whether inhibitory feedback onto MBON-11 contributes to the regulation of aggression, we selectively reduced GABA receptor function in these neurons and quantified behavioral outputs. We knocked down the ionotropic GABA_A_ receptor Rdl (resistant to dieldrin) (*66*), as well as individual γ-aminobutyric acid type B (GABA_B_) receptor subunits, specifically in MBON-11 neurons. Rdl knockdown significantly shortened lunge latency (Fig. 7A), produced a modest increase in total lunges (Fig. 7B), and significantly elevated boxing frequency (Fig. 7, C and D), with 78% of male pairs exhibiting high-intensity fighting. Silencing GABA_B_ receptors did not produce a similar increase in aggression (fig. S6). These findings suggest that fast ionotropic GABAergic signaling via Rdl in MBON-11 neurons plays a key role in limiting the escalation of aggressive behavior, whereas metabotropic GABA_B_ signaling contributes to a lesser extent.

**Fig. 8.**
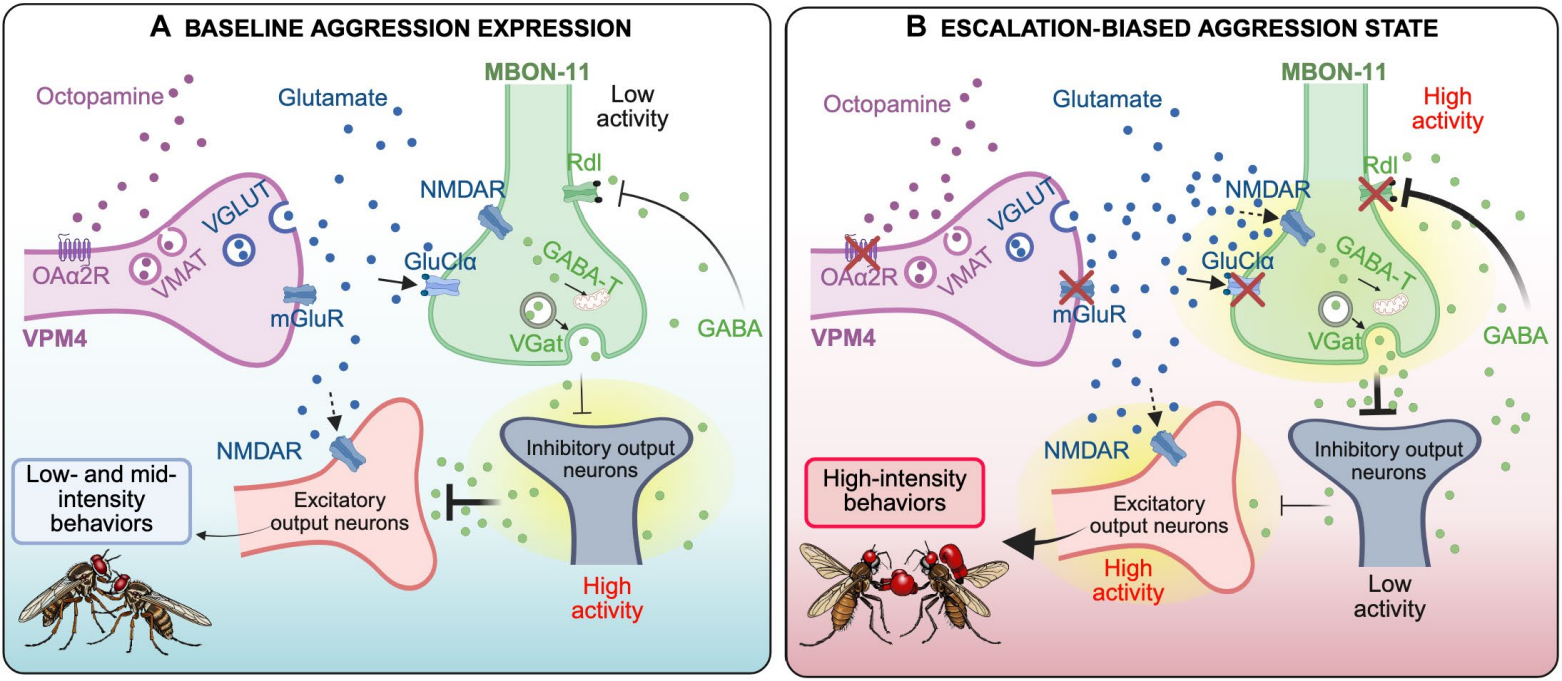
Layered inhibition model controlling aggression intensity. (**A**) Under basal VPM4 activity, glutamatergic signaling through GluClα is proposed to reduce MBON-11 activity, limiting MBON-11 GABA release. In this state, downstream inhibitory neurons remain active, thereby constraining excitatory output pathways and restricting behavior to low- and mid-intensity aggression such as lunges. (**B**) When circuit activity shifts toward increased MBON-11 output, elevated GABA release is proposed to inhibit downstream inhibitory neurons, resulting in disinhibition of excitatory pathways and promoting high-intensity behaviors such as boxing. Increased MBON-11 activity is also associated with Rdl-dependent inhibitory feedback, which limits sustained MBON-11 output. Our data support a model in which transmitter-specific feedback mechanisms (OAα2R and mGluR), GluClα-dependent inhibition of MBON-11, and Rdl-mediated inhibitory signaling act at multiple levels to constrain escalation. While the anatomical sources of these signals are not directly resolved, these mechanisms are consistent with a layered inhibitory organization that regulates aggression intensity. Dashed lines indicate hypothetical or untested pathways, including potential direct signaling from VPM4 to downstream output neurons and possible glutamatergic signaling via NMDARs in MBON-11. Created in BioRender. Trannoy, S. (2026), https://biorender.com/02wi5af.

Single-cell and bulk RNA sequencing datasets reveal that MBON-11 neurons express Rdl, and endogenous Rdl-smV5 tagging confirms localization of the receptor to MBON-11 dendrites (*59*), consistent with a role in synaptic inhibition. The source of GABAergic input acting through Rdl will require additional experiments other than receptor manipulation; however, one possibility is that MBON-11 neurons engage in local self-inhibition via autapses, synapses a neuron forms onto itself (*24*, *67*), which could stabilize their synaptic activity. Alternatively, MBON-11 activity may recruit distinct GABAergic neurons that provide circuit-level feedback inhibition. Regardless of the source, our findings support a role for Rdl-dependent GABAergic signaling in limiting MBON-11 activity, thereby constraining sustained approach drive and preventing excessive escalation. Disruption of this inhibitory control promotes persistent high-intensity aggression, potentially by reducing retreat (Fig. 8).

## DISCUSSION

Aggression is a crucial social behavior observed across species that provides access to resources, territory, and mates. However, when expressed at excessive levels, aggression in humans can be life-threatening, increase the risk of developing psychiatric disease in those affected, and impose notable economic burdens to society. Because of the risks of escalated aggression, it is critical to determine the control mechanisms that initiate and constrain aggression. Our study uncovers a previously uncharacterized control pathway that modulates the intensity of male aggressive behavior in *Drosophila*. Specifically, our findings are consistent with a layered inhibitory circuit organization where OA-glutamate cotransmitting VPM4 neurons regulate the activity of the downstream GABAergic neuron, MBON-11 (Fig. 8). This layered inhibition includes (i) transmitter-specific presynaptic feedback mechanisms that regulate OA and glutamate release, (ii) GluClα-mediated inhibition of MBON-11, and (iii) inhibitory feedback loops that gate MBON-11 output. These findings are integrated in a conceptual model (Fig. 8) in which these mechanisms act together to constrain the escalation of aggressive behavior. While our data do not directly resolve all circuit architecture or neurotransmitter sources, these mechanisms are consistent with converging feedback processes that restrain escalation while preserving flexibility in behavioral responses.

Within this circuit, VPM4 is well positioned to couple resource valuation to aggression. In feeding circuits, OA released from VPM4 terminals activates OAMB on sugar-responding gustatory neurons to amplify their output, and VPM4 boutons respond most strongly when sweetness is paired with hunger state, indicating integration of external resource cues with internal motivational signals (*16*, *17*). Building on this framework, our activation and transmitter manipulation experiments extend the role of VPM4 from feeding to aggression, supporting a model in which VPM4 may influence escalation thresholds when resource value and motivational drive justify the metabolic costs of high-intensity fighting.

Beyond its valuation role, this transmitter-specific control may be evolutionarily conserved, as evidenced in the locus coeruleus, where NE and glutamate cotransmission to the central amygdala engages a defined circuit that suppresses feeding during fear responses (*19*). In *Drosophila*, we find an analogous principle within a single cotransmitting neuron: Presynaptic OAα2R and mGluR feedback in VPM4 constrains escalation, as knockdown of either receptor increases boxing without altering baseline lunging. In parallel, VPM4-derived OA suppresses male-male courtship during fights, indicating that VPM4 can coordinate competitive and reproductive drives through separable output modes. Together, these results suggest that VPM4 contributes to dual behavioral control by deploying OA through distinct signaling modes, likely including synaptic transmission for aggression-related output and broader extrasynaptic signaling for courtship suppression. Supporting the broader concept that monoamine output can be partitioned across discrete release sites and signaling modes, OA signaling influences female fertility in *Drosophila* (*68*), and noradrenaline transmission can occur at defined synapses in vertebrate circuits (*69*). Overall, these results support the idea that cotransmission paired with transmitter-specific feedback provides a flexible mechanism for regulating competing behaviors across contexts.

Although glutamate is generally known as an excitatory neurotransmitter, it can also mediate inhibition through ligand-gated chloride channels such as GluClα (*70*). Prior work demonstrated that optogenetic activation of VPM4 suppresses odor-evoked responses in MBON-11, indicating inhibitory synapses from VPM4 to MBON-11 (*17*, *18*). Our findings support the conclusion that this glutamatergic inhibition is mediated by GluClα receptors in MBON-11. Similar inhibitory roles for GluClα have been reported in the *Drosophila* olfactory system, where glutamatergic local neurons inhibit the firing of GABAergic interneurons through GluClα (*70*). Reducing GluClα-mediated inhibition in MBON-11, via GluClα knockdown or direct MBON-11 activation, was sufficient to increase high-intensity boxing without affecting mid-intensity lunging, consistent with GluClα functioning as a brake on MBON-11 activity within a value-processing circuit. While increasing glutamatergic output from VPM4 modulates aggression, the inhibitory nature of VPM4 to MBON-11 signaling is supported by GluClα-dependent effects and functional imaging (*18*), rather than by VGLUT manipulations. The use of inhibitory rather than excitatory glutamatergic signaling, likely originating from VPM4, suggests the importance of having a brake mechanism within a value-processing neuron.

Whether MBON-5 functionally regulates MBON-11 cannot be determined from our behavioral data alone and would require direct tests of functional connectivity between these neurons. Nevertheless, increasing glutamatergic output from MBON-5/6 did not reproduce the aggression escalation observed with VPM4 manipulations, suggesting that glutamatergic inputs to MBON-11 have source-specific functional consequences. Such specificity may arise from spatial segregation at multiple levels. First, glutamatergic synapses from distinct presynaptic partners, such as VPM4 and MBON-5, may target different subcellular compartments of MBON-11. Second, postsynaptic receptor composition may be heterogeneously distributed, with VPM4-derived glutamate preferentially engaging GluClα-enriched domains, thereby mediating inhibition, whereas other inputs may engage different receptor populations.

Our findings converge with previous studies that identified the MBON-11 GABAergic interneuron as providing feed-forward inhibition to downstream targets that respond to aversive and rewarding odor stimuli. Perisse *et al.* (*20*) demonstrated that MBON-11 signaling promotes approach behavior by inhibiting avoidance circuits. On the basis of our results and previous reports, we propose a model in which the MBON-11 neuron receives inhibitory glutamatergic input from VPM4 to control aggressive escalation. This hierarchical arrangement, where glutamate inhibits a GABAergic output neuron, creates multiple layers of inhibitory control. Thus, the GABAergic output from a single MBON-11 neuron can orchestrate both odor-guided decisions and aggression escalation through context-specific inhibition of its downstream targets. Consistent with state dependence, VGAT knockdown alone does not alter baseline aggression but prevents the increased boxing induced by MBON-11 activation, indicating that MBON-11 GABA output becomes behaviorally relevant when these neurons are recruited. In this line, recent work in the mammalian cortex has revealed how distinct inhibitory cell populations create multilayered control of information flow, from layer-specific targeting in the prefrontal cortex to the orchestration of dendritic integration in pyramidal neurons (*71*).

MBON-11 forms more than 100 autapses (*24*, *67*) and expresses the ionotropic Rdl GABA receptors (*57*). Removing Rdl increases escalation, consistent with a role for fast inhibitory feedback in keeping MBON-11 below the threshold that triggers high-cost fighting. Negative feedback mediated by OAα2R and mGluR in VPM4 and Rdl in MBON-11 likely operates within a physiological range, acting as a brake to constrain escalation under normal conditions. Genetic manipulations that elevate transmitter availability could shift the balance between drive and feedback inhibition, thereby biasing the circuit toward escalation. Consistent with state dependence, VGAT knockdown alone does not alter baseline aggression but prevents the increased boxing induced by MBON-11 activation, indicating that MBON-11 GABA output becomes behaviorally relevant when these neurons are recruited (Fig. 7). Similar to findings in mammalian cortical circuits, where autaptic connections in fast-spiking interneurons provide rapid neuromodulator-sensitive feedback control, our results align with computational predictions that autaptic feedback serves as a dynamic gain control mechanism (*72*). This arrangement may allow MBON-11 to integrate circuit-level inputs with local autoregulation, creating a tunable threshold for escalation that could be adjusted by neuromodulatory signals. Given OA’s role as a neuromodulator, determining whether and how it modulates MBON-11 activity will be an important next step.

Although our study centers on male-specific escalation circuits, females also engage in competitive interactions, albeit with distinct motor patterns such as headbutting behavior instead of boxing (*13*, *73*). Whether the VPM4-MBON-11 circuit also governs female aggression or has been repurposed for other behaviors, such as feeding, remains an open question. Investigating whether VPM4-derived glutamate and OA also inhibit MBON-11 in females could help clarify how the same molecular components are differentially deployed across sexes to potentially support sexually dimorphic behaviors (*74*).

## MATERIALS AND METHODS

### Fly stocks

All *D. melanogaster* strains used in this study are listed as follows: Canton-S wild-type (Cs, Kravitz’s laboratory, Harvard Medical School, Boston, MA), MB112C-Gal4 (MBON-11) (BDSC no. 68263), MB113C-Gal4 (VPM4) (BDSC no. 68264), MB298B-Gal4 (MBON-5) (BDSC no. 68309), UAS-dTrpA1 (Kravitz’s laboratory, Harvard Medical School, Boston, MA), UAS-VMAT^GFP^ (Steven Stowers), UAS-VGLUT (BDSC no. 81119), UAS-GAD1myc-HA (BDSC no. 93606), UAS-OAα2R-RNAi (BDSC no. 50678), UAS-TβH-RNAi (BDSC no. 27667), UAS-mGluR-RNAi (BDSC no. 34872), UAS-NMDAR1-RNAi (BDSC no. 25941), UAS-NMDAR2-RNAi (BDSC no. 40846), UAS-GluClα-RNAi (BDSC no. 53356), UAS-GABA-T-RNAi (BDSC no. 54993), UAS-VGAT-RNAi (VDRC no. 103586), UAS-Rdl-RNAi (BDSC no. 52903), UAS-GABA-B-R1-RNAi (BDSC no. 51817), UAS-GABA-B-R2-RNAi (BDSC no. 50608), UAS-GABA-B-R3-RNAi (BDSC no. 50622), OAa2R-FRT-F3-20XV5 (Steve Stowers), OAa2R-40XFLAG (Steve Stowers), UAS-mCherry (BDSC no. 27392), OAα2R-lexA (BDSC no. 52743), lexAop-nucRFP (BDSC no. 32205), RSRT-STOP-RSRT-9XV5-vGAT (BDSC no. 94894), and *w;; GluCl*α*-KD-KDRT-STOP-KDRT-smGdP-10xV5/TM6B* (gift of S. Lawrence Zipursky).

### Fly housing

All flies were reared on standard cornmeal-based fly food. Unless otherwise noted, all flies were raised and housed in incubators at 20°C and 50% relative humidity and a 12:12-hour light:dark cycle with white light-emitting diode strips. Flies used for behavioral assays were isolated at the late pupa stage by placing them in plastic vials containing 1 ml of standard fly food. Isolated flies with the UAS-dTrpA1 reporter were reared at 20°C for 7 days before aggression assays, while the other flies with UAS-RNAi, UAS-VGLUT and UAS-VMAT were reared at 20°C for 4 days and then transferred to 25°C for the remaining 3 days before the aggression assays.

### Aggression and intermale courtship assays

Aggression assays were performed with 7-day-old flies between 09:00 and 12:00, which corresponds to Zeitgeber time 0 (ZT0) to ZT3. For experiments with UAS-dTrpA1–containing males, arenas were placed in an incubator at 29°C (or 20°C for control experiments in figs. S1 and S4) the day before the assays. For experiments with UAS-RNAi, UAS-VGLUT, and UAS-VMAT, arenas were placed in an experimental room at 25°C the day before the assays. In all assays, flies were inserted inside the arenas for 10 min of acclimation before the start of the experiment.

Behavioral assays were performed in sliding arenas (22-mm diameter by 16-mm height) previously described in (*75*) in Trannoy’s lab. A food cup (13 by 6 mm) was placed in the center of the arena, filled with standard fly food and a drop of rehydrated yeast (Sigma-Aldrich, cat. no. YSC2) in the center. An opaque divider was inserted from the cover to separate the arena in two equal sizes. Males were inserted by negative geotaxis on each side of the arena. After 10 min of acclimatization, the divider was removed, and flies could start interacting. Social interactions were recorded for 35 min with Basler Pylon 5.x cameras. The first meeting, the number of lunges, and the number of boxing events were scored semiautomatically with BORIS version 8.21.5 (*76*). The numbers of lunges and boxing events were scored for 5 min after the first lunge. If no lunge occurred for 15 min after the first meeting, we scored that this was a fight without lunges.

Results presented in Figs. 1 (I to L) and 2 and fig. S2 were performed in the divider chamber described in (*75*) in Certel’s lab. Behavior chambers were assembled on clear agarose (Sigma-Aldrich, cat. no. 05040) with a yeast/sucrose–based (Sigma-Aldrich, cat. no. 9378) food top and placed on top of a light-emitting diode light pad (AGPtek). Pairs of 5- to 7-day-old, socially naïve adult males were aspirated into the divided chambers and left for a period of at least 24 hours to allow for recovery from anesthetization. Fly movements were tracked using CalTech FlyTracker 1.0.5 (available for download at https://kristinbranson.github.io/FlyTracker/) (*77*), and lunges were subsequently quantified using the Janelia Automatic Animal Behavior Annotator (JAABA) (*78*). The lunge classifier (Certel_lungeClassifier.jab) was designed in JAABA (referencing human-scored data) and used to detect lunges from individual flies. Annotated frames were postprocessed in JAABA with the internal postprocessing filter set at 0.06, a value that provided the best signal-to-noise ratio a posteriori for lunge classification. An additional postprocessing filter was applied in MATLAB (MathWorks, www.mathworks.com/) using JAABA postprocessed files in combination with tracking data to eliminate misclassified lunges detected at a distance of two or more fly body lengths. The total number of lunges was scored for a period of 30 min after the first lunge using a custom MATLAB script (analysis.m). The time between the beginning of the video recording and the first lunge was used for calculating the latency to lunge. Boxing events were scored for a period of 10 min after the first lunge. Intermale courtship was defined as the number of unilateral wing extensions (singing) followed by additional courtship behaviors (licking, abdomen bends, repeated wing extensions, etc.) (*79*).

### Immunochemistry

Adult male dissected brains were fixed in 4% paraformaldehyde (Electron Microscopy Sciences) for 25 min and labeled using a modification of protocols previously described (*10*). The following primary antibodies were used in this study: rat anti-V5 (Bio-Rad, orb256445), rabbit anti-GFP (Invitrogen, G10362), mouse anti-GFP (Invitrogen, A11120), rabbit anti-mCherry (Abcam, 167453), rat anti-HA 3F10 (Roche Millipore Sigma, C755C30), anti-bruchpilot (Developmental Hybridoma Studies Bank, nc82), anti-mGluR (monoclonal, gift from I. Sinning), anti-dVGLUT (gift from B. McCabe), and anti-NMDAR2 (source unknown). The following secondary antibodies were used: donkey anti-mouse Alexa Fluor 647 (Life Technologies, A31571), goat anti-rabbit Alexa Fluor 488 (Invitrogen, A11008), and goat anti-rabbit Alexa Fluor 594 (Invitrogen, A11037). Secondary antibodies conjugated to Alexa Fluor 488, Alexa Fluor 594, or Alexa Fluor 647 (Molecular Probes) were used at a concentration of 1:200. Labeled brains were mounted in Vectashield (Vector Labs, no. H1000). Images were collected on a Zeiss 880 laser scanning confocal microscopy system and processed using ImageJ [National Institutes of Health (NIH), https://imagej.net/software/fiji/] and Adobe Photoshop (Adobe, CA, www.adobe.com/).

### Statistical analysis

All graphs were generated with Prism (GraphPad Software) and Adobe Illustrator CS6 (Adobe). GraphPad Prism 8.2.1 (for Windows, GraphPad Software, San Diego, CA, www.graphpad.com) and RStudio 2021.09.1+372 were used for statistical analysis. Outliers were identified with Grubb’s test (α = 0.05). These extreme values were removed from the analysis. Most of our datasets did not pass the normality test (Shapiro-Wilk); we therefore used the nonparametric Kruskal-Wallis statistical test. Dunn’s multiple comparisons test was used as a post hoc test to compare which groups significantly differed from the others. A Mann-Whitney test was performed in the case of two category comparisons. Proportions were compared on RStudio (R Core Team, 2020, www.r-project.org/) by using a generalized mixed model with binomial distribution, followed by Tukey’s post test and Fisher’s test with a Holm-Bonferroni post test.
