# Supplementary material for: An octopamine-glutamate cotransmitting neuron gates aggressive escalation through layered inhibition in Drosophila
Source: Sci Adv. 2026 Aug 14;12(33):eaec4153. doi: 10.1126/sciadv.aec4153 (PMC13475639; doi:10.1126/sciadv.aec4153)

Science Advances
AAAS

# Supplementary Materials for

## An octopamine-glutamate cotransmitting neuron gates aggressive escalation through layered inhibition in *Drosophila*

Antoine Prunier *et al.*

Corresponding author: Sarah J. Certel, sarah.certel@umontana.edu;
Séverine Trannoy, severine.trannoy@utoulouse.fr

*Sci. Adv.* **12**, eaec4153 (2026)
DOI: 10.1126/sciadv.aec4153

**This PDF file includes:**

Figs. S1 to S6

**Fig. S1. Experimental males do not display high-intensity aggression at 20 °C**

(**A**) When experiments are performed at non-permissive temperature, the latency to lunge is not affected (Kruskal-Wallis, H=1.669 p=0.434). (**B**) VPM4/+ control males give less lunges than VPM4/dTrpA1 (Kruskal-Wallis H=13.71, p=0.001, Dunn's test p=0.0008). (**C**) The number of boxing events is not different between groups (Kruskal-Wallis H=2.091, p=0.351).

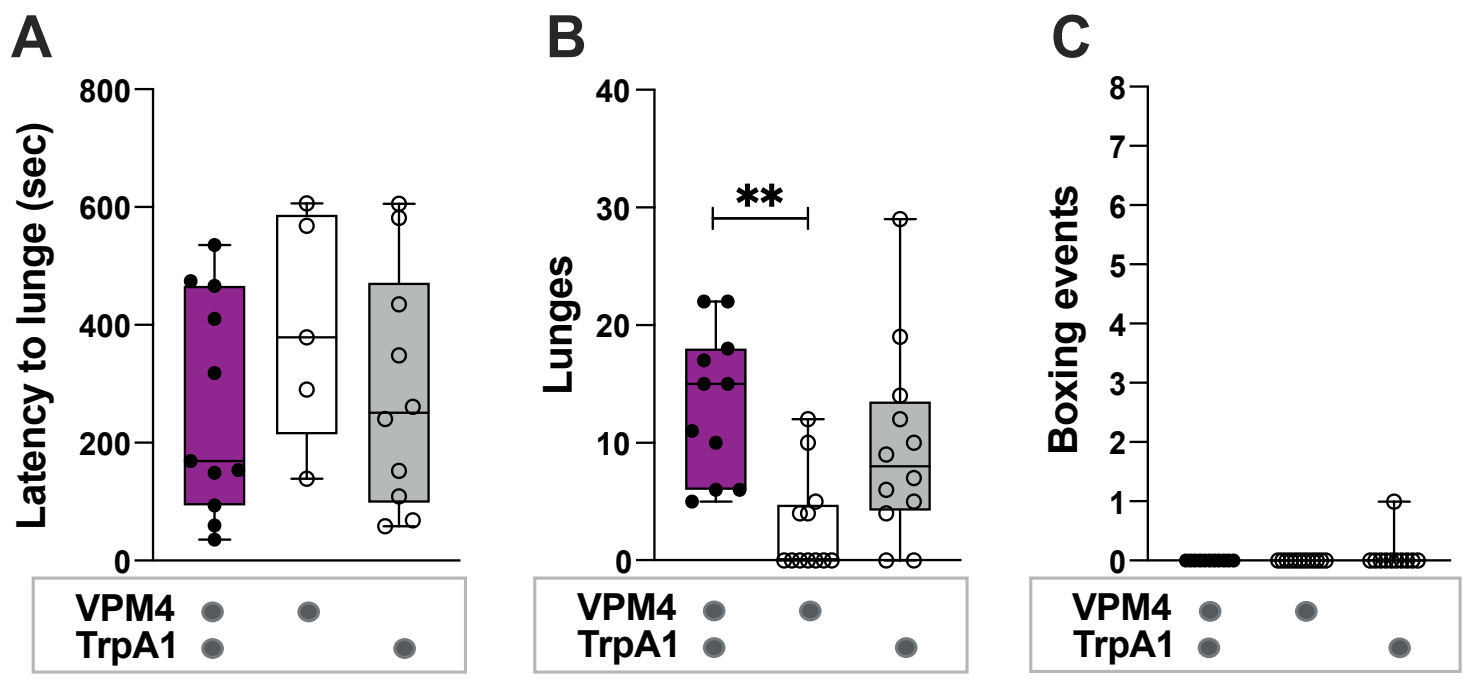

**Fig. S2. NMDAR1 or NMDAR1/2 downregulation does not alter male aggression**

**(A)** *MB123C-splitGal4* and *NMDAR2-LexA* together with UAS-6XGFP and LexAop-6XmCherry reporters demonstrated NMDAR2 (red) is expressed in MBON-11 neurons (green). (**B**) Down-regulating expression of NMDAR1 doesn't lead to changes in latency to lunge. However, this parameter is different for *MBON-11/+* genotype (Kruskal-Wallis H=12.24, p=0.002, Dunn's test with *MBON-11>NMDAR1*$^{KD}$ p=0.0276 and with *+/NMDAR1*$^{KD}$ p=0.0027). (**C**) The number of lunges is also increased in MBON-11/+ control males (Kruskal-Wallis H=10.67, p=0.0048, Dunn's test with *MBON-11>NMDAR1*$^{KD}$ p=0.018 and with *+/NMDAR1*$^{KD}$ p=0.0098). **(D)** Downregulation of NMDAR2 in MBON-11 (*MBON-11>NMDAR2*$^{KD}$) increases the latency to lunge compared to *+/NMDAR2*$^{KD}$ control (Kruskal-Wallis, H=9.01, p=0.011, Dunn's test p=0.011) but not to *MBON-11/+ control* (p=0.097). **(E)** The number of lunges is reduced in *MBON-11/NMDAR2*$^{KD}$ males compared to compared to *MBON-11/+* control (Kruskal-Wallis H=7.005, p=0.030, Dunn's test p=0.041), but not to *+/NMDAR2*$^{KD}$ control (p=0.091). (**F**) Co-expressing RNAi against both NMDAR1 and NMDAR2 receptors in MBON-11 doesn't impact the number of lunges compared to controls (Kruskal-Wallis H=11.82, p=0.008).

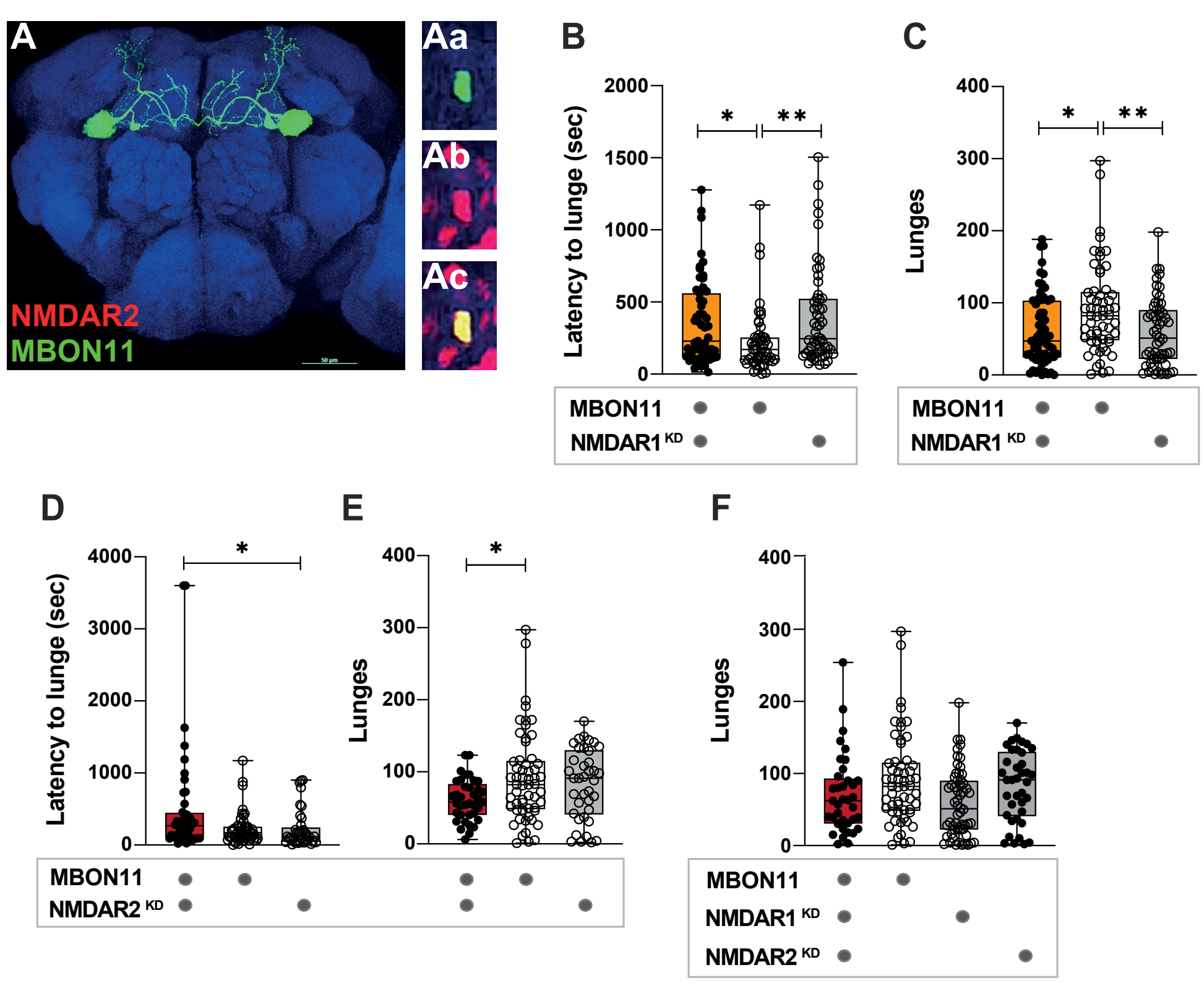

**Fig. S3. Inhibitory control of MBON-11 through GluClα does not rely on MBON-5 inputs**

**(A)** Increasing glutamate release from MBON-5 (*MBON-5/6>VGLUT*) reduced the latency to lunge compared to UAS control (Kruskal-Wallis H=8.487, p=0.014, Dunn's test p=0.015) but not to GAL4 control (p=0.153). **(B)** Similarly, the number of lunges is increased only compared to UAS control (Kruskal-Wallis H=8.488, p=0.014, Dunn's test p=0.020) but not to GAL4 control (p=0.079). **(C)** However, increasing glutamatergic release from MBON-5 doesn't trigger boxing events (Kruskal-Wallis H=4.051, p=0.132). **(D)** The boxing frequency is not significantly increased compared to controls (Fisher's Exact Test for Count Data p=0.069).

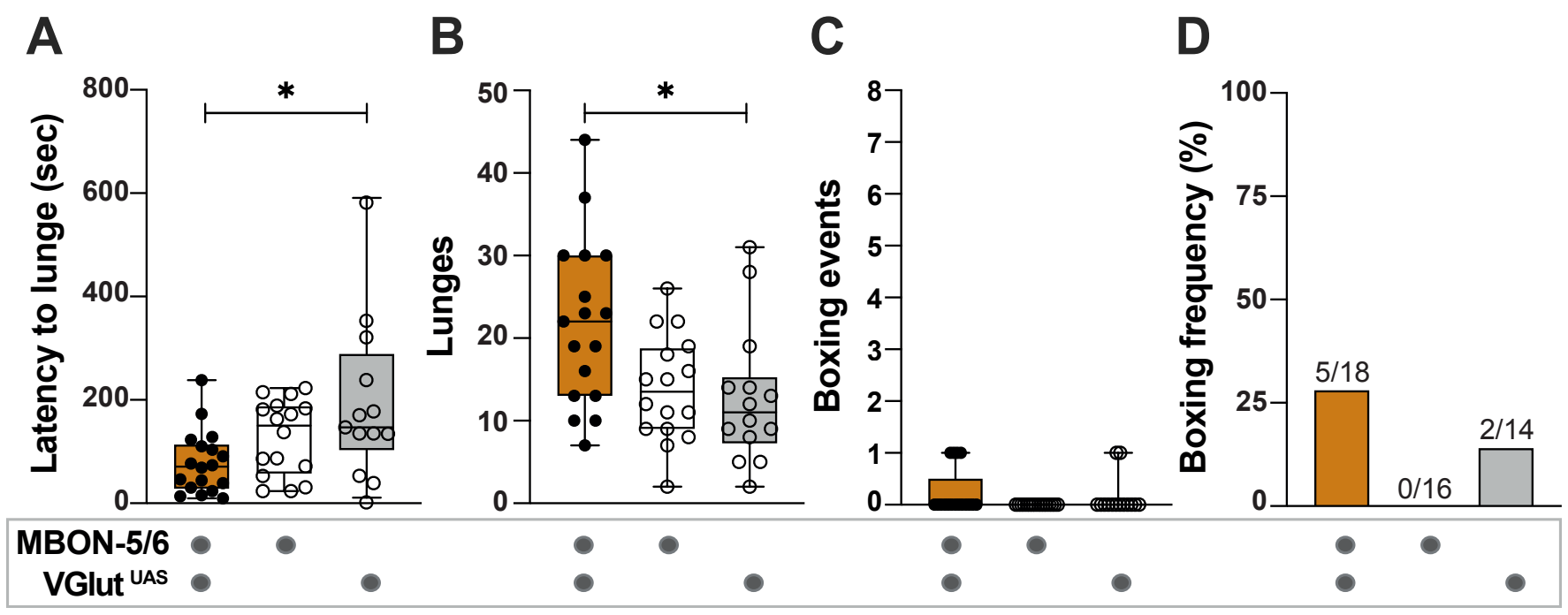

**Fig. S4. Low temperature (20 °C) suppresses high-intensity aggression in experimental males**
(**A**) When performing experiments at low temperature, no differences in the number of lunges is observed (Kruskal-Wallis H=1.939, p=0.379). (**B**) The number of boxing events is not different between groups (Kruskal-Wallis H=0.365, p=0.833), neither is (**C**) the boxing frequency (Generalized linear model with a binomial distribution=0.193, Df=2, p=0.908).

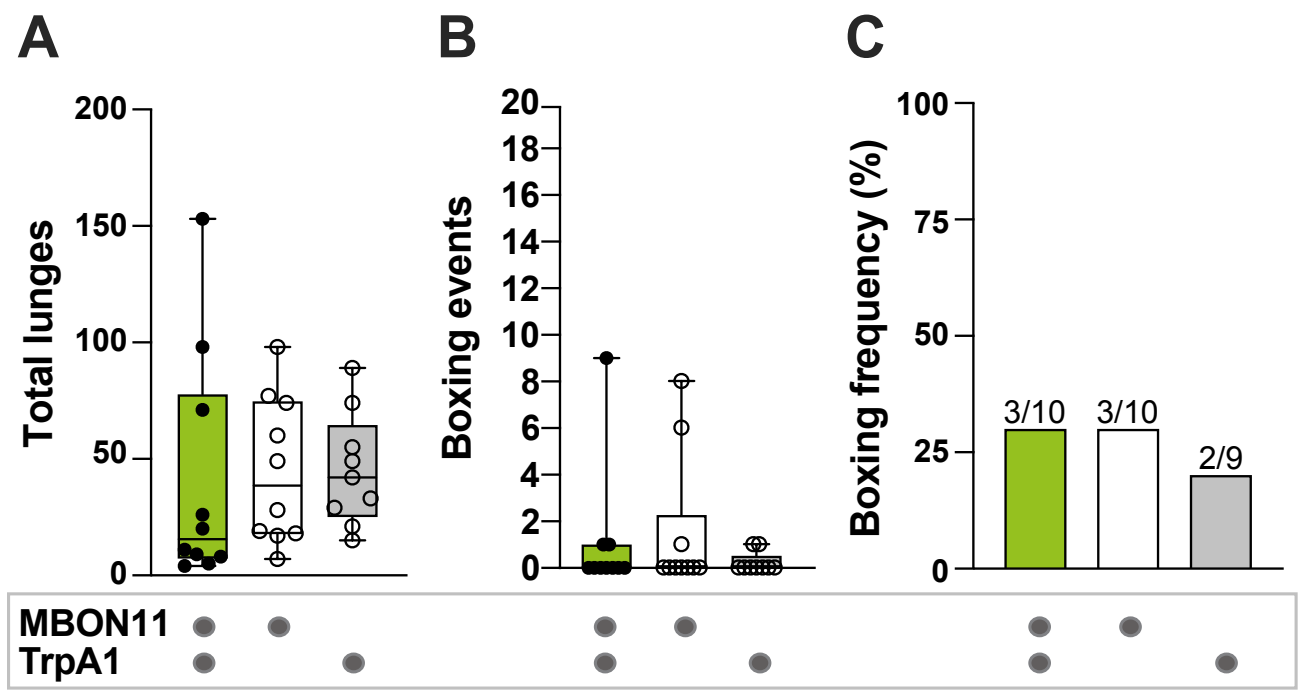

**Fig. S5. Over-expression of GAD1 doesn't induce high-intensity male-male aggression.**

(**A**) Increasing expression of GAD1 enzyme within MBON-11 doesn't impact the number of lunges. However, the GAL4 control group tends to display more lunges (Kruskal-Wallis H=8.187, p=0.017). (**B**) The number of boxing event is not increased (Kruskal-Wallis H=4.807, p=0.09), **(C)** nor the boxing frequency (Generalized linear model with a binomial distribution=2.801, Df=2, p=0.246).

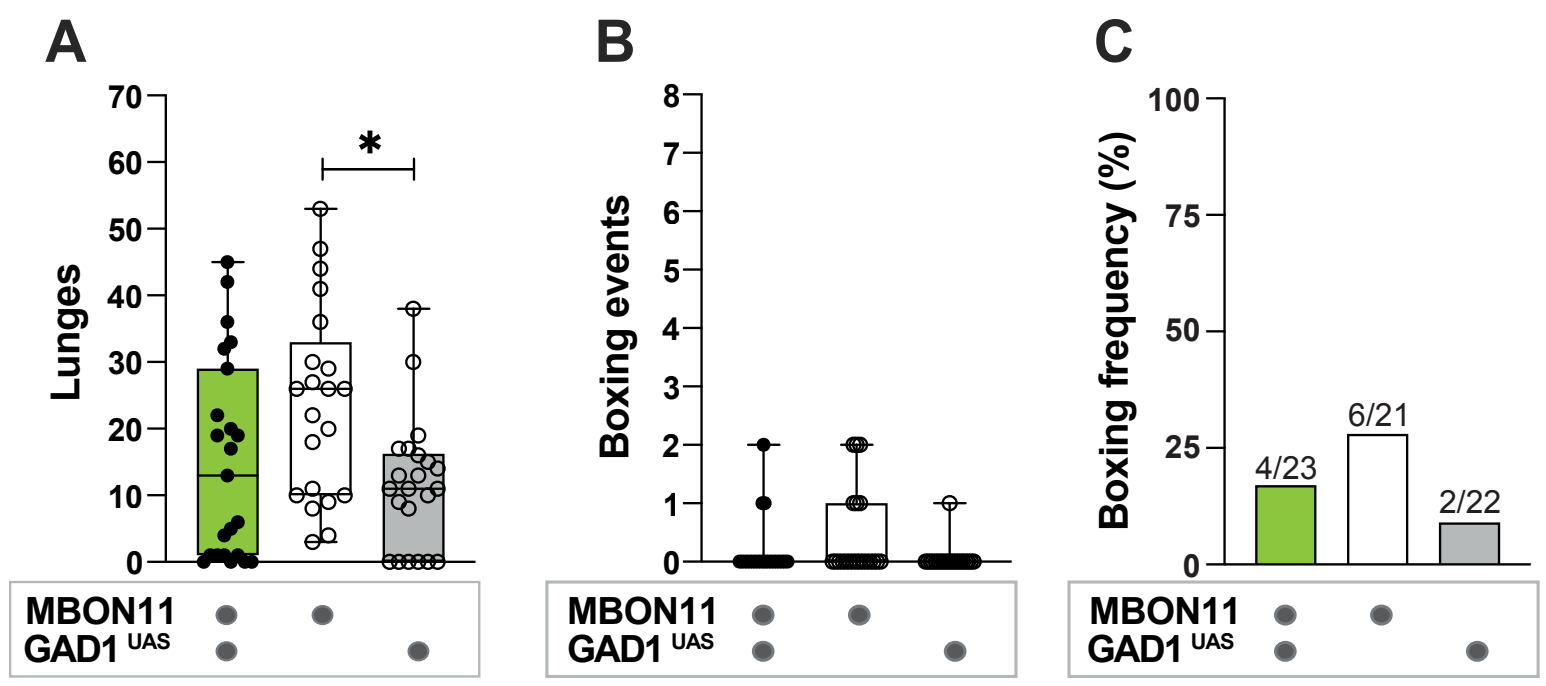

**Fig. S6. Slow GABAergic feedback via GABAB-R3 tends to enhance aggression**

**(A)** The number of boxing event was statistically increased in *MBON-11>GABA*$_B$*-R3*$^{KD}$ males compared to genetic controls (Kruskal-Wallis H=25.10, p=0.0003, Dunn's test with *MBON-11/+* p=0.006 and with *+/GABA*$_B$*-R3*$^{KD}$ p=0.006). No differences were found for GABA$_B$-R1 and R2. **(B)** However, the boxing frequency is not significantly increased when downregulating GABA$_B$-R3 in MBON-11 compared to genetic controls (Fisher's Exact Test for Count Data p=0.0005; Holm-Bonferroni post-test with *MBON-11/+* p=0.059, and with *+/GABA*$_B$*-R3*$^{KD}$ p=0.053).

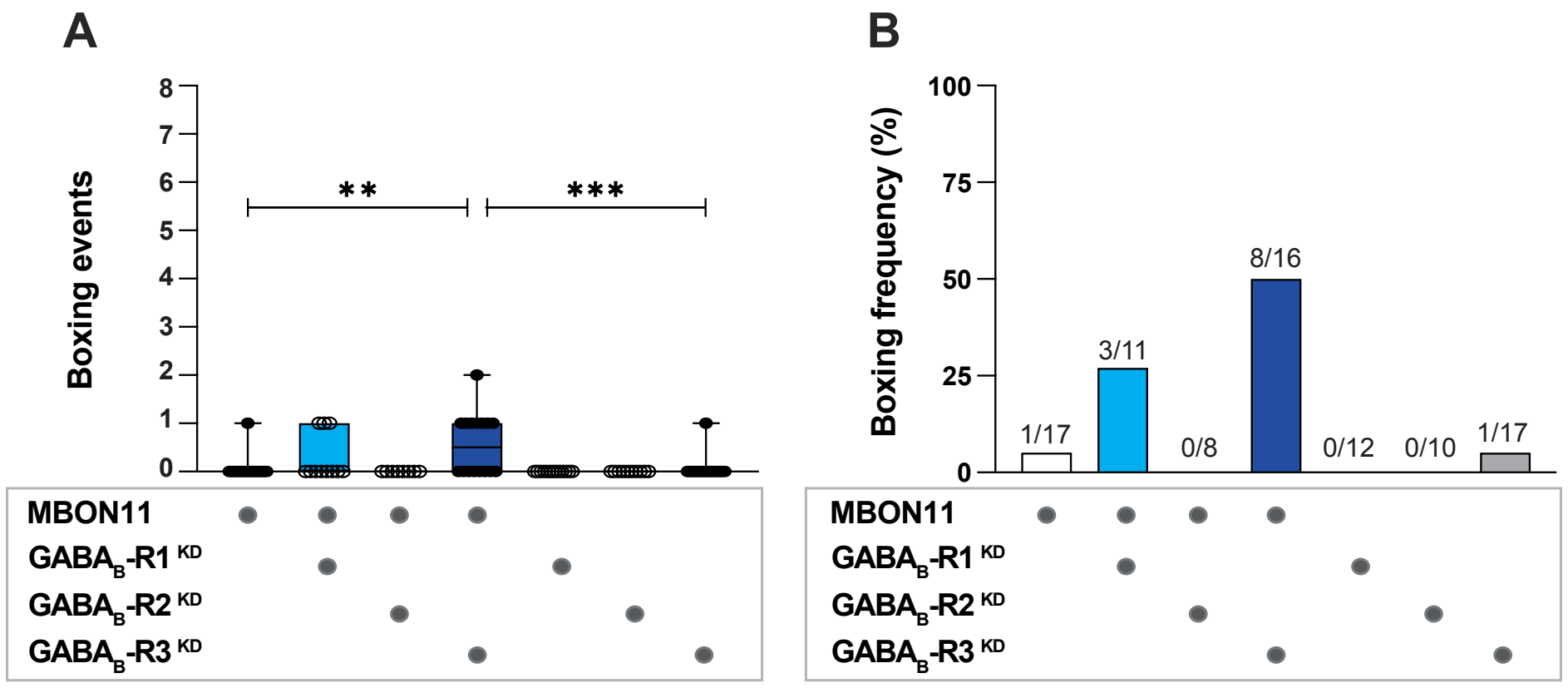

Supplement: Supplementary file 1 [file sciadv.aec4153_sm.pdf]
